# Secondary overactive bladder syndrome with restless legs syndrome following cerebral infarction: report of 2 cases

**DOI:** 10.1186/s12877-025-05724-z

**Published:** 2025-03-25

**Authors:** Ying Cui, Qiang Huang, Yitong Du, Lin Wang, Shiya Wang, Wenlu Zhao, Houzhen Tuo

**Affiliations:** Department of Neurology, Beijing Friendship Hospital, Capital Medical University, No. 95 YongAn Rd, Beijing, 100050 China

**Keywords:** Overactive bladder syndrome, Restless legs syndrome, Restless bladder syndrome, Cerebral infarction, Dopaminergic agonists

## Abstract

**Background:**

Previous research has commonly regarded overactive bladder syndrome (OAB) and restless legs syndrome (RLS) as distinct disorders in terms of their underlying causes, clinical diagnosis, and treatment approaches. However, there is evidence of an overlap in the occurrence of these two conditions following cerebral infarction(CI). Specifically, restless bladder syndrome (RBS), a subtype of RLS, exhibits symptoms similar to those of OAB. Consequently, further investigation is warranted to better understand the relationship between these two disorders.

**Case presentation:**

In this report, we present the cases of two patients who presented with both OAB following CI, along with RLS. Following administration of oral pramipexole, both nocturia and RLS exhibited prompt and efficient alleviation.

**Conclusions:**

There may exist a shared pathological foundation between certain RLS cases and OAB. In instances where patients exhibit evident OAB symptoms following CI and concurrently experience RLS, it is advisable to prioritize the administration of dopaminergic agonists over M receptor inhibitors and β_3_ agonists. Furthermore, it is plausible that the RBS or a subset of RLS cases could potentially be classified as a form of OAB, although this correlation has yet to be definitively established.

## Introduction

Overactive bladder syndrome (OAB) is defined by the International Continence Society (ICS) as a syndrome presenting with symptoms related to urinary storage. It is characterized by a sense of urgency to urinate, with or without involuntary leakage, and typically accompanied by increased frequency of urination, in the absence of urinary tract infection or any other identifiable organic pathology [1].

Findings from a cross-sectional survey conducted in Europe indicate that the prevalence of OAB among individuals aged 50 years and older was 11.8%, with slightly higher rates observed in women (12.8%) compared to men (10.8%), and a positive correlation with advancing age [2]. In a study conducted by Tikkinen et al. [3], it was found that 31% of patients with nocturia (those who urinated more than twice at night) had OAB. This finding suggests that OAB is prevalent among those with heightened nocturia, ultimately impacting their quality of sleep. The diagnosis of OAB relies on a comprehensive patient history assessment and physical examination, and both routine urine examination and urinary ultrasound (including ultrasound determination of the post-void residual (PVR)  volume) are essential. When a specific pathology is suspected, further evaluation tools such as pathological examination, urine cytology, and Kidney Ureter Bladder (KUB) imaging are required [4].

Restless legs syndrome (RLS) is a sensory-motor disorder that often features unpleasant sensations in the legs, such as burning, pain, or stiffness, resulting in a strong desire to move the legs. These symptoms are most noticeable at night and at rest, and can be partially or completely relieved by activity or massage [5]. The etiology of RLS commonly includes factors such as iron deficiency and renal insufficiency [6]. The occurrence of RLS following cerebral infarction(CI) is not infrequent, as evidenced by a reported incidence of 10% by Chandan et al. [7]. In a prior investigation conducted by our research team, we observed a prevalence of 5.33% for RLS secondary to lacunar CI. Furthermore, we identified a significant association between RLS and infarcts located in the corona radiata, basal ganglia, and pons [8].

A cross-sectional survey of 500 post-stroke patients showed that the prevalence of OAB in patients was 28%. Additionally, the probability of OAB after CI and cerebral hemorrhage events was similar. It is worth noting that a significant proportion (73%) of OAB cases were not treated by urologists [9]. These findings suggest a potential association between CI and OAB and RLS through specific pathomechanisms. However, further investigation is required to understand the relationship between these two disorders. In this report, we present two patients who experienced both OAB and RLS after CI. Notably, treatment with oral pramipexole resulted in rapid and effective relief of both nocturia and RLS symptoms.

## Report of 2 patients

### Patient 1

**Fig. 1:**
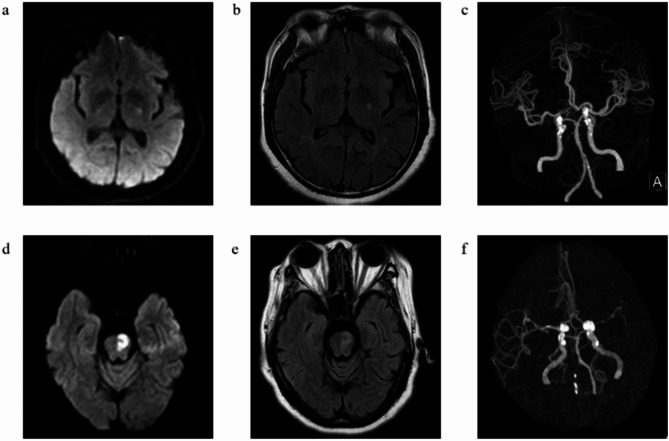
Site of CI and intracranial vascular images in 2 patients **a-b**. DWI imaging and FLAIR imaging showed that the left thalamic punctate high signal of case 1 was suggestive of a new infarction; **c.** Intracranial CTA images at the same level of case 1 demonstrated his calcified plaques in the siphon segments of the internal carotid arteries bilaterally; **d-e.** DWI imaging and FLAIR imaging showed that the left ventral posterolateral pons revealed patchy high signal of case 2, which indicated the site of new infarction; **f.** Intracranial CTA images of case 2 hinted multiple intracranial stenosis of the patient, with severe stenosis of the right vertebral artery

A 68-year-old male patient was admitted to the hospital in October 2021 due to the sudden onset of right limb weakness accompanied by slurred speech and sleepiness persisting for a duration of 3 days, with a medical history of hypertension, diabetes mellitus, and hypothyroidism. He denied any previous or familial history of RLS. Admission examination: He presented with mild drowsiness, dysarthria, a shallow nasolabial groove on the right side, right deviation of tongue extension, muscle strength grade IV on the right limbs, grade V on the left, decreased pinprick sensation on the right limbs, normal sensation on the left side, ataxia on the right limbs, no ataxia on the left side, Babinski’s sign was observed as positive on the right side and negative on the left side. The National Institutes of Health Stroke Scale (NIHSS) score was recorded as 7. The head Magnetic resonance imaging(MRI) revealed long T1 and T2 signals in the left thalamus, along with the high Diffusion weighted imaging(DWI) signal, the high Fluid attenuated inversion recovery(FLAIR) signal (Fig. 1a-b), and the low Apparent Diffusion Coefficient(ADC) signal. The Computed tomography angiography(CTA) showed severe narrowing of the lumen in the ophthalmic segment of the right internal carotid artery (Fig. 1c). He was diagnosed with acute CI in the left thalamus and left frontal lobe. The patient was prescribed aspirin combined with clopidogrel for anti-platelet aggregation, atorvastatin for lipid-lowering and plaque stabilization, ginkgo biloba for improving circulation, butylphthalide to promote the development of collateral circulation, as well as appropriate medication to lower blood pressure and blood sugar levels.

On the third day after the onset of the disease, the patient reported experiencing nocturnal discomfort in both lower limbs, primarily characterized by pain. These episodes occurred 3–5 times per night, each lasting several minutes, and were noticeably alleviated by physical activity or massage. Concurrently, the patient experienced an increase in nocturia, urinating 5 times per night, which negatively impacted his sleep. Upon admission and subsequent reexamination, the patient’s blood routine was normal. His urine analysis did not detect the presence of leukocytes, and the bladder ultrasound did not reveal any obvious abnormalities. There was no evident enlargement of the prostate, and the PVR volume was 86 mL. Frequent nocturia caused by urinary tract infections and or high PVR was ruled out. The patient scored 0 on the Hamilton depression scale and 1 on the Hamilton anxiety scale. Based on the diagnostic criteria for OAB and RLS established in 2014, a diagnosis of OAB combined with RLS secondary to acute CI was made. Given an International Restless Legs Syndrome Study Group (IRLSSG) score of 20, the patient was prescribed pramipexole at a dosage of 0.125 mg nightly. Following a two-day course of medication, the patient experienced notable relief from RLS, accompanied by a significant decrease in the frequency of nocturia. Subsequently, the patient continued to maintain a dosage of 0.125 mg nightly. By the two-week follow-up after discharge, the frequency of nocturia had decreased to twice per night. Furthermore, there was a marked improvement in legs pain symptoms compared to the pre-treatment period, as evidenced by a 3-point score on the IRLSSG scale. With no deterioration of symptoms observed over a six-month follow-up period, pramipexole was gradually discontinued. Currently, the drug has been discontinued for over six months. The patient’s follow-up results indicate a nocturia frequency of less than or equal to two occurrences per night, an IRLSSG score of 3, and a NIHSS score of 1.

### Patient 2

A 64-year-old female patient was admitted to the hospital in February 2021 due to the sudden onset of right limb weakness persisting for five days. Her medical history includes hypertension, diabetes mellitus, and residual effects from a previous CI (memory loss and right-limb weakness). She denied any previous or familial history of RLS. Upon physical examination, the patient presented with dysarthria, grade II muscle strength in the right upper limb, grade III muscle strength in the right lower limb, and grade V muscle strength in the left limbs. The patient exhibited diminished pinprick sensation and ataxia on the right limbs, accompanied by a positive right pathology sign. The NIHSS score was recorded as 7. Head MRI: long T1 and T2 signals in the left pontine, along with the high DWI signal, the high FLAIR signal (Fig. 1d-e), and the low ADC signal. The CTA revealed moderate to severe stenosis in the bilateral internal carotid artery siphon segment, as well as in the intracranial segment of the right vertebral artery and the lumen of the left middle cerebral artery (Fig. 1f). The diagnosis was acute CI (left pontine). Her admission blood and urine routine were normal. After admission, she was given aspirin to inhabit platelet aggregation, atorvastatin lipid-lowering and stabilizing, ginkgo biloba to improve circulation, butylphthalide to establish collateral circulation.

On the 9th day of hospitalization, the patient’s weakness in the right limbs worsened and she presented with somnolence. The latest head MRI imaging: The DWI signal of the original left pontine infarction was weakened, but the signal of the right pontine was high, suggesting the possibility of another pontine infarction. Simultaneously, the patient experienced frequent nocturia, occurring 10 times per night—an average frequency of approximately once per hour, as well as each time the volume of urine was small(under 200 mL). A repeat blood biochemistry test showed no anemia, electrolyte disorders, or renal function abnormalities. There were no leukocytes in the urine. Her urinary ultrasound was normal and 68 mL of residual urine was stored in the bladder. Upon re-examination, the patient exhibited periodic limb movements in the right lower limb during wakefulness, specifically dorsiflexion of the toes and foot, with intervals of 30–40 s between episodes. However, her legs discomfort was very mild, barely noticeable. Her IRLSSG score was 8. Taking into account the patient’s clinical symptoms, as well as laboratory and imaging findings, a diagnosis of OAB and RLS secondary to acute cerebral pontine infarction was made. Oral pramipexole at a dosage of 0.125 mg per night resulted in a reduction in the frequency of nocturnal urination to 2–3 times per night. Subsequent follow-up visits were conducted at 2 weeks, 6 months, and 2 years after discharge, respectively, revealing that the number of nocturia episodes remained consistent at 2–3 times per night, with no reports of leg discomfort and improved sleep quality. The patient is currently maintained on pramipexole 0.125 mg nightly, which effectively manags the symptoms of OAB and RLS.

The scores of the two patients before and after pramipexole treatment were displayed (Table 1), demonstrating significant improvement in nocturia and RLS symptoms. The head MRI and CTA results were presented concurrently (Fig. 1).

**Table 1:** Summary of patient OAB and RLS symptoms

		pre-reatment	post-reatment
Case1	Nocturia	4–5 times /night	≤ 2 times/night
	IRLSSG score	20	3
	NIHSS score	7	1
	HAMA score	1	1
	HAMD score	0	2
Case2	Nocturia	10 times /night	2–3 times /night
	IRLSSG score	8	4
	NIHSS score	7	4
	HAMA score	7	7
	HAMD score	9	4

OAB, Overactive bladder; RLS, Restless legs syndrome; IRLSSG, International restless legs syndrome study group; NIHSS, National institutes of health stroke scale; HAMA, Hamilton anxiety scale; HAMD, Hamilton depression scale

## Discussion and conclusion

The etiology of OAB is complex and is considered to be a multifactorial symptomatic syndrome arising from various underlying pathophysiological mechanisms [10]. Expert consensus recommends the use of M-receptor antagonists and β_3_-adrenergic receptor agonists as the first-line treatment for OAB, which results in an approximate 70% improvement rate [11]. This outcome implies the existence of an etiology beyond vegetative dysfunction.

Abnormalities in bladder afferent signaling pathways are thought to be the primary pathogenesis of OAB. Detrusor overactivity (DO) has been identified as a direct cause of the symptoms of urinary urgency and frequency in OAB [12]. In addition to peripheral factors such as uroepithelial and subepithelial dysfunction in the afferent pathway, increased urethral-derived stimuli, and pharmacological neurotoxicity injury [13–15], the attenuation of the inhibitory effect of the central nervous system (CNS) can indirectly lead to heightened excitability of afferent nerve impulses. Related studies have indicated that both acute and chronic cerebral ischemic lesions may be associated with DO-related OAB [16]. Specifically, acute ischemia in the anterior medial frontal lobe with its inferior pathway, the basal ganglia, and the nucleus accumbens is common regions where OAB occurs [17, 18].

The two patients reported in this study exhibited increased nocturia following CI, with clinical symptoms aligning with an OAB diagnosis after ruling out other urinary tract causes such as infection and obstruction. The patient in Case 1 exhibited a more pronounced diurnal rhythm of increased nocturia, concomitant with RLS. This clinical presentation suggests a preferential approach to the treatment of RLS. Following treatment, the patient’s symptoms improved, supporting the hypothesis that the diagnosis was consistent with RLS following CI, in conjunction with a variant form of RLS, termed Restless Bladder Syndrome (RBS). This syndrome includes discomforting sensations in the lower abdomen and perineum, which are relieved by urination and predominantly occur during nighttime. This particular variant effectively responds to dopaminergic medication. Notably, the patient in case 2 did not exhibit prominent symptoms of restless legs but rather presented with isolated RBS. Antelmi et al. [19] were the first to report two cases of RBS. In both cases, urinary frequency, urgency, and increased nocturia were the only symptoms, without restless legs. The patient in this case, who was treated with dopaminergic medication, exhibited a significant reduction in OAB. Consequently, RBS may represent an unrecognized etiology of OAB.

Previous studies have demonstrated that CNS impairment can induce symptoms of bladder irritation [20, 21]. Previous studies have commonly treated OAB and RLS as distinct disorders in terms of pathogenesis, clinical diagnosis, and treatment approaches. However, a combination of both types of symptoms has been reported. A Finnish cohort study found that RLS had a positive prediction rate of approximately 42% in OAB [3].Additionally, a cross-sectional study involving 18 women with RLS revealed a prevalence of 67% for the co-occurrence of OAB and RLS [22].These aforementioned studies indicate that the comorbidity of OAB and RLS is not merely coincidental. Regrettably, neither of the two studies provided an explanation for this phenomenon, and few studies have mentioned the simultaneous presence of these conditions. Our investigation identified a potential intersection of these phenomena. It is essential to distinguish patients with RLS from those with OAB. Administering targeted, first-line treatment with RLS medication may help alleviate the patients’ OAB.

In conclusion, OAB should not be regarded as an independent ailment, but rather as a spectrum of subtypes influenced by different mechanisms (some possibly corresponding to an overlooked form of RBS). This finding suggests that dopaminergic agonists should be prioritized for patients with this specific subtype of OAB. This study further elucidates the etiological factors underlying OAB and introduces novel therapeutic approaches for patients who do not respond to conventional treatments. These insights hold considerable significance for guiding clinical work.

Furthermore, it is necessary to ascertain the degree of overlap between OAB and RLS. Additionally, it is crucial to determine the efficacy of dopaminergic agents in alleviating OAB in patients unresponsive to M-receptor inhibitors, both with and without comorbid RLS. These inquiries necessitate further investigation through clinical cohort studies.

## Data Availability

All data needed to evaluate the conclusions are present in the paper.

## Abbreviations

OAB: Overactive bladder syndrome
RLS: Restless legs syndrome
RBS: Restless bladder syndrome
CI: Cerebral Infarction
IRLSSG: International restless legs syndrome study group
NIHSS: National institutes of health stroke scale
HAMA: Hamilton anxiety scale
HAMD: Hamilton depression scale
MRI: Magnetic resonance imaging
CTA: Computed tomography angiography
FLAIR: Fluid attenuated inversion recovery
DWI: Diffusion weighted imaging
ADC: Apparent diffusion coefficient
